# Steroid-Responsive Demyelinating Myelopathy

**DOI:** 10.51894/001c.165962

**Published:** 2026-07-31

**Authors:** Razan Mohanna, Vashudaven Mittal, Kamal Khalil, Hassan Cheema

**Affiliations:** 1 DMC - Sinai Grace

## 105

Inflammatory demyelinating disorders affecting the nervous system represent a diverse group of diseases that may overlap in their features. We present the case of a 56-year-old woman who experienced subacute progressive neurological deficits.

The patient had a medical history positive for hypertension, hyperlipidemia, chronic obstructive pulmonary disease, and cervical myelomalacia. In September 2025, she started having distal paresthesia in both upper and lower extremities. Within one week, she experienced weakness in both upper and lower extremities, accompanied by gait instability and falls. Her weakness was slightly more pronounced on the left side. She reported transient urinary incontinence. Later and at her presentation, she was unable to ambulate independently.

Neurologic examination demonstrated: hyporeflexia, bilateral weakness, with preserved sensation and a mild predominance on the left side, normal cranial nerve examination, and unsteady gait. Cerebrospinal fluid analysis revealed elevated protein with low lymphocytic cell counts, matched serum and CSF oligoclonal bands, and a normal Immunoglobulin G index, consistent with albumin-cytologic dissociation. Autoimmune and paraneoplastic panels were negative.

An MRI of the brain revealed a peripherally enhancing right parietal lesion with extension into the splenium of the corpus callosum, along with involvement of the ependymal involvement. The cervical spine MRI revealed cord expansion with intramedullary signal abnormality and enhancement at the C5–C6 level; these findings were more consistent with an inflammatory demyelinating process. At a later stage, electromyography and nerve conduction studies revealed an acquired demyelinating polyneuropathy within the acute and chronic inflammatory demyelinating polyneuropathy spectrum.

Based on the combined clinical and paraclinical findings, the patient was started on intravenous methylprednisolone, followed by a tapering dose of oral prednisone. She showed rapid and significant neurological improvement within days. After her discharge, she went to a rehabilitation center. During her three-month follow-up, she reported near complete recovery.

This case highlights the diagnostic challenges associated with conditions that involve demyelination of the central and peripheral nervous system. The patient’s response to corticosteroid therapy supports the presence of an underlying inflammatory, immune-mediated process. This case highlights the importance of combining clinical and paraclinical findings as well as clinical progress in patients with subacute neurological symptoms.

